# Efficacy of platinum agents for stage III non-small-cell lung cancer following platinum-based chemoradiotherapy: a retrospective study

**DOI:** 10.1186/s12885-022-09441-3

**Published:** 2022-03-29

**Authors:** Eriko Miyawaki, Hirotsugu Kenmotsu, Yasushi Shintani, Ikuo Sekine, Takehito Shukuya, Koichi Takayama, Akira Inoue, Isamu Okamoto, Katsuyuki Kiura, Kazuhisa Takahashi, Nobuyuki Yamamoto, Tomoya Kawaguchi, Etsuo Miyaoka, Ichiro Yoshino, Hiroshi Date

**Affiliations:** 1 Division of Thoracic Oncology, Shizuoka Cancer Center, 1007 Shimonagakubo, Nagaizumi-cho, Sunto-gun, Shizuoka, 411-8777 Japan; 2 Department of General Thoracic Surgery, Osaka University Graduate School of Medicine, Osaka, 565-0871 Japan; 3 Department of Medical Oncology, Faculty of Medicine, University of Tsukuba, Tsukuba, 305-8576 Japan; 4 Department of Respiratory Medicine, Juntendo University Graduate School of Medicine, Tokyo, 113-8431 Japan; 5 Department of Pulmonary Medicine, Kyoto Prefectural University of Medicine, Kyoto, 602-8566 Japan; 6 Department of Palliative Medicine, Tohoku University School of Medicine, Miyagi, 980-8574 Japan; 7 Research Institute for Diseases of the Chest, Graduate School of Medical Sciences, Kyushu University, Fukuoka, 812-8582 Japan; 8 Department of Allergy and Respiratory Medicine, Okayama University Hospital, Okayama, 700-8558 Japan; 9 Internal Medicine III, Wakayama Medical University, Wakayama, 641-8509 Japan; 10 Department of Respiratory Medicine, Graduate School of Medicine, Osaka Metropolitan University, Osaka, 545-8586 Japan; 11 Department of Mathematics, Tokyo University of Science, Tokyo, 162-8601 Japan; 12 Department of General Thoracic Surgery, Graduate School of Medicine, Chiba University, Chiba, 260-8677 Japan; 13 Department of Thoracic Surgery, Graduate School of Medicine, Kyoto University, Kyoto, 606-8507 Japan

**Keywords:** Cytotoxic chemotherapy, Platinum-based chemotherapy, Second-line setting, Single-agent chemotherapy, Survival

## Abstract

**Background:**

Platinum-based chemoradiotherapy is the standard treatment for unresectable stage III non-small-cell lung cancer (NSCLC). However, few studies have evaluated the efficacy of subsequent chemotherapy for relapsed NSCLC following platinum-based chemoradiotherapy. This study aimed to evaluate the efficacy of platinum-doublet chemotherapy as a second-line treatment for patients with unresectable stage III NSCLC.

**Methods:**

We retrospectively evaluated patients with unresectable stage III NSCLC treated with cytotoxic chemotherapy following platinum-based chemoradiotherapy who were registered in a nationwide registry NSCLC database. Patients were divided into the platinum-doublet chemotherapy (platinum) group and single-agent chemotherapy (non-platinum) group based on the type of second-line chemotherapy.

**Results:**

The platinum group (*n* = 119) showed significantly better overall survival (OS) than the non-platinum group (*n* = 201) (median OS: 21.5 vs. 10.5 months, hazard ratio [HR]: 0.54, 95% confidence interval [CI]: 0.40–0.73, *p* < 0.001). OS from the beginning of chemoradiotherapy was also significantly better in the platinum group than in the non-platinum group (median OS: 34.9 vs. 21.8 months, HR: 0.58, 95% CI: 0.43–0.79, *p* = 0.001). In the multivariate analysis, platinum-doublet chemotherapy as second-line therapy, female sex, clinical stage IIIA, and duration of ≥ 8.6 months from the beginning of first-line therapy to the beginning of second-line therapy were associated with significantly better OS.

**Conclusion:**

Platinum-doublet chemotherapy as a second-line therapy may prolong survival in unresectable stage III NSCLC patients following platinum-based chemoradiotherapy. Thus, re-administration of platinum agents may be a promising treatment for unresectable stage III NSCLC treated with platinum-based chemoradiotherapy.

## Background

Lung cancer is the leading cause of cancer-related deaths worldwide. Approximately one-third of patients with non-small-cell lung cancer (NSCLC) present with locally advanced nonmetastatic disease equivalent to stage III disease at the time of diagnosis [1]. Platinum-based chemoradiotherapy (CRT) with curative intent is the standard treatment modality for patients with unresectable stage III NSCLC [2, 3]. However, most patients show disease progression after CRT, with a median progression-free survival (PFS) of 10–21 months [4–7]. Durvalumab, a programmed death-ligand 1 (PD-L1) inhibitor, was recently proven to be effective for consolidation therapy after platinum-based CRT and thus was approved as a new standard therapy for unresectable stage III NSCLC. However, many patients still show disease progression, with a 12-month PFS rate of approximately 50–60%, and consequently need second-line treatment [8, 9].

Non-platinum cytotoxic single-agent chemotherapy (e.g., docetaxel or pemetrexed) is currently one of the standard treatments in the second-line setting after first-line platinum-based chemotherapy for advanced NSCLC patients not harboring oncogenic drivers, such as mutations in the epidermal growth factor receptor (*EGFR*) gene or translocations of the　anaplastic lymphoma kinase gene. However, patients treated with single-agent chemotherapy as a second-line treatment still have a poor prognosis, with a response rate of < 10% and a median survival of only 6–8 months [10–12].

A pooled analysis of 10 studies on patients with advanced NSCLC who received platinum rechallenge showed that the objective response rate (ORR) to platinum doublets as a second-line therapy was 27.5%, with a median PFS of 3.9 months and a median overall survival (OS) of 8.7 months [13]. These results suggest that platinum doublets as subsequent chemotherapy may be an effective treatment option. Given that the previous studies mostly included patients with stage IV disease, there is limited data on the efficacy of subsequent platinum-based chemotherapy for relapsed stage III NSCLC patients previously treated with platinum-based CRT. Therefore, this study aimed to evaluate the efficacy of platinum-doublet chemotherapy as a second-line treatment for patients with unresectable stage III NSCLC previously treated with platinum-based CRT.

## Methods

### Study design and patients

This was a retrospective study on unresectable stage III NSCLC patients treated with cytotoxic chemotherapy following platinum-based CRT, registered in the Japanese Joint Committee of Lung Cancer Registry (JJCLCR) database. The JJCLCR has gathered the medical records of lung cancer patients from 314 Japanese educational institutions. The registry was opened between January 1, 2012, and December 31, 2012, and follow-up was completed on April 30, 2016. Participating institutions performed registration by accessing the website set up by the JJCLCR, as described in the registry survey [14].

The inclusion criteria for the registry were (1) pathological or cytological diagnosis of any type of lung cancer at a participating institution and (2) confirmation of diagnosis between January 1, 2012, and December 31, 2012. The staging was based on the Union for International Cancer Control version 7 [15], and the disease stage was assigned based on the findings of plain chest radiography, computed tomography (CT) of the chest and abdomen, positron emission tomography or bone scintigraphy, and CT or magnetic resonance imaging of the cranium.

The inclusion criteria for this retrospective cohort study were as follows: clinical stage IIIA or IIIB NSCLC, treatment with platinum (carboplatin or cisplatin)-based concurrent CRT as a first-line therapy, and cytotoxic chemotherapy administered as second-line therapy. We excluded patients harboring *EGFR* mutations and those receiving *EGFR*-tyrosine kinase inhibitors, such as gefitinib and erlotinib, as a second-line chemotherapy regardless of *EGFR* mutation status. Patients treated with chemotherapy including bevacizumab and non-platinum doublets were also excluded.

The included patients were divided into two groups according to the second-line chemotherapy regimen. The platinum group involved patients treated with platinum-doublet chemotherapy, while the non-platinum group involved those treated with non-platinum single-agent chemotherapy. The registry followed the ethical guidelines for epidemiologic studies. This study was approved by the Institutional Review Board of Osaka University Medical Hospital, where the registry office is located. Because this was a retrospective study, patient consent was waived, and anonymity was ensured.

### Data collection and response evaluation

Data on age, sex, smoking status, the existence of respiratory comorbidities, Eastern Cooperative Oncology Group performance status (ECOG PS), histology, clinical stage at the start of CRT, type of platinum agent and radiation dose used in the first-line CRT, response to CRT, and duration from the start of the first-line CRT to the date of starting second-line chemotherapy were extracted from the master database. Clinicopathological profiles, OS, and tumor response were evaluated. The tumor response was assessed according to the Response Evaluation Criteria in Solid Tumors (RECIST) version 1.1 [16].

### Statistical analyses

Patient characteristics were compared both descriptively and using Fisher’s exact test for categorical variables and the Mann–Whitney U test for continuous variables. OS was measured as the interval between the start of second-line chemotherapy and death or censored at the last follow-up date. Survival curves were plotted using the Kaplan–Meier method and compared using standard log-rank tests. Univariate and multivariate analyses were performed using Cox proportional hazard models to calculate the hazard ratios (HRs) and corresponding 95% confidence intervals (CIs) and to examine differences with respect to OS from the start of second-line chemotherapy in each group. Variables included age, sex, ECOG PS, smoking status, histology, clinical stage, the existence of respiratory comorbidity, tumor response to first-line therapy, and the duration from the start of the first-line CRT to the start of the second-line chemotherapy. The median value was used as a cut-off for the duration from the start of the first-line CRT to the start of the second-line chemotherapy. Information on the reasons for the termination of the first-line CRT was not available in this registry. Thus, subgroup analyses stratified by the duration from the start of the first-line CRT to the start of the second-line chemotherapy were performed, with a cut-off of 3 months to exclude the effect of patients terminating first-line platinum-based therapy early for reasons other than disease progression. All statistical analyses were performed using the SPSS software program, version 23.0 (SPSS Inc., Chicago, Illinois) for Windows. *P*-values (two-sided) less than 0.05 were considered statistically significant.

## Results

### Patient characteristics

**Fig. 1:**
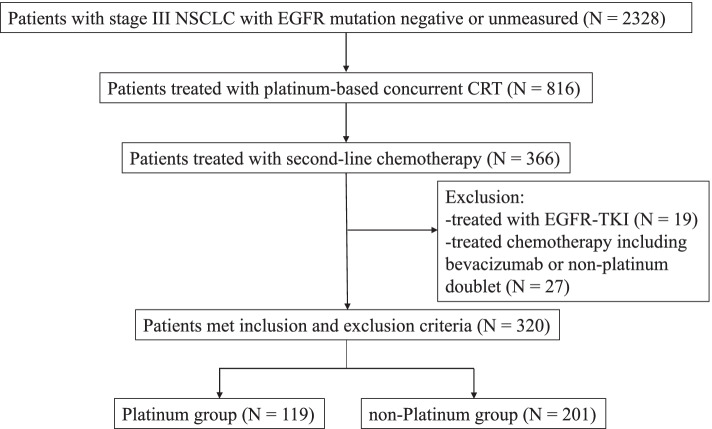
Patient inclusion flowchart

Of the 320 patients included, 119 (37%) and 201 (63%) patients received platinum-doublet chemotherapy (platinum group) and single-agent chemotherapy (non-platinum group) in the second-line setting, respectively (Fig. 1). The median age was 64 years, 87% of the patients were male, and 94% were current or former smokers. Approximately 40% of patients had respiratory comorbidities, including pulmonary emphysema in 102 (32.5%) and interstitial lung disease in 14 (4%) patients. The most common histological types were adenocarcinoma and squamous cell carcinoma. The characteristics of the patients at the start of platinum-based concurrent CRT are shown in Table 1. There were no significant between-group differences in clinicopathological characteristics, such as age, sex, smoking status, respiratory comorbidity, and ECOG PS. There were also no significant differences in the type of platinum agents or radiation doses used for first-line platinum-based CRT (Table 2). The ORR of first-line CRT was not significantly different between the two groups (65.5% in the platinum group vs. 68.7% in the non-platinum group, *p* = 0.62). The median time from the start of the first-line CRT to the start of the second-line chemotherapy was 8.6 months (range, 0.5–46.2 months). The interval between first-line CRT and second-line chemotherapy was significantly shorter in the platinum group than in the non-platinum group (median time: 6.6 vs. 8.7 months, *p* < 0.01). The interval was < 3 months in 26% of patients in the platinum group and 4.5% of those in the non-platinum group.

**Table 1:** Patient characteristics at the start of first-line chemoradiotherapy

Characteristics	Platinum group		non-Platinum group			*P*-value
	n	%	n		%	
Overall	119		201			
Age (years)						
Median (range)	63	(33—81)	65		(44—80)	
≥ 75	8	6.7	15		7.5	1.0
< 75	111	93.3	186		92.5	
Sex						
Female	18	15.1	25		12.4	0.50
Male	101	84.9	176		87.6	
Smoking status						
Current	68	57.1	115		57.2	1.0
Former	44	37.0	75		37.3	
Never	7	5.9	11		5.5	
Respiratory comorbidity						
Yes	43	36.1	78		38.8	0.72
No	76	63.9	123		61.2	
ECOG PS						
0	66	55.5	100		49.8	0.41
1	49	41.2	97		48.3	
2	4	3.4	4		2.0	
Histology						
Adenocarcinoma	51	42.9	85		42.3	0.99
Squamous cell carcinoma	53	44.5	89		44.3	
Other	15	12.6	27		13.4	
Clinical stage (UICC-TNM classification, 7th edition)						
IIIA	60	50.4	95		47.3	0.64
IIIB	59	49.6	106		52.7	

*ECOG PS* Eastern Cooperative Oncology Group performance status, *UICC* Union for International Cancer Control

All *P* values were calculated using the Fisher’s exact test

**Table 2:** Treatment and efficacies of first-line chemoradiotherapy

	Overall		Platinum group		non-Platinum group		*P*-value
	*n* = 320		*n* = 119		*n* = 201		
	n	%	n	%	n	%	
Platinum							
CDDP	189	59.1	68	57.1	121	60.2	0.21
CBDCA	129	40.3	49	41.2	80	39.8	
Unknown	2	0.6	2	1.7	0	0	
Radiation dose							
≥ 60 Gy	271	84.7	96	80.7	175	87.1	0.10
< 60 Gy	47	14.7	23	19.3	24	11.9	
Unknown	2	0.6	0	0	2	1.0	
Response to chemoradiotherapy							
ORR (%)		67.5		65.5		68.7	0.62
Duration from the start of first-line therapy to the start of second-line therapy							
Median (range), (months)	8.6	(0.5–46.2)	6.6	(0.5–46.2)	8.7	(0.5–38.0)	< 0.01

*CDDP* cisplatin, *CDBCA* carboplatin, *ORR* objective response rate, *Gy*, gray

*P* values were calculated using the Fisher’s exact test for categorical variables and with Mann–Whitney U test for continuous variables

### Survival outcomes

**Fig. 2:**
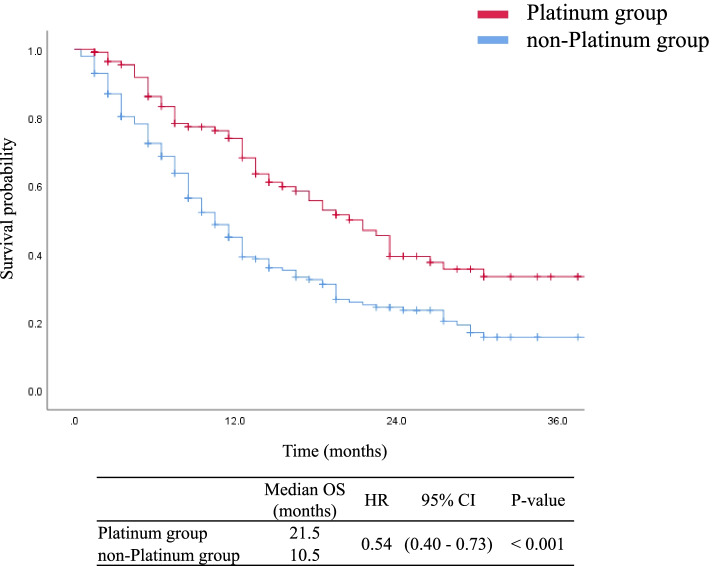
Kaplan–Meier curves of overall survival from the beginning of second-line chemotherapy until death or the last follow-up in patients treated with platinum-based chemotherapy and single agent in second-line setting. Tick marks represent data censored at the last time the patient was known to be alive. Abbreviations: CI, confidence interval; HR, hazard ratio; OS, overall survival

**Fig. 3:**
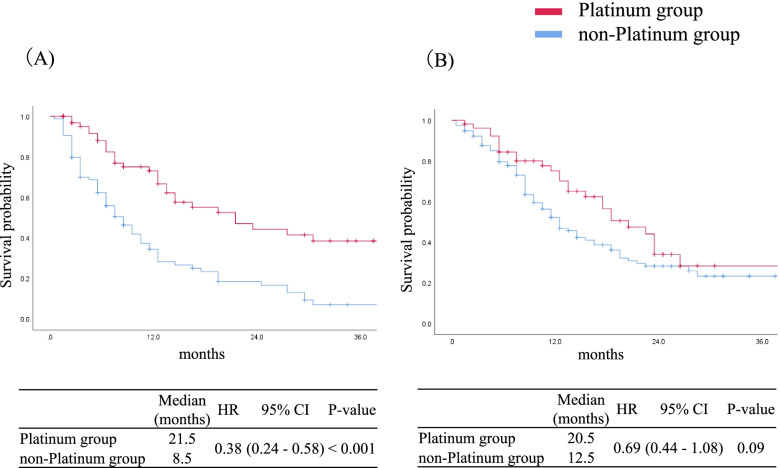
**A** Kaplan–Meier curves of overall survival from the beginning of second-line chemotherapy until death or the last follow-up in patients treated with platinum-based chemotherapy and single agent in the second-line setting in the subgroup of patients with an interval of < 8.6 months between the start of first-line CRT and the start of second-line chemotherapy and (**B**) in the subgroup of patients with an interval of ≥ 8.6 months between the start of first-line CRT and the start of second-line chemotherapy. Tick marks represent data censored at the last time the patient was known to be alive. Abbreviations: CI, confidence interval; CRT, chemoradiotherapy; HR, hazard ratio; OS, overall survival

At the time of data cut-off (April 30, 2016), the median follow-up duration from the start of second-line chemotherapy for censored cases was 14.0 months (range, 1.5–42.5 months). Of the 320 patients, 198 (61.8%) had died by the time of data cut-off. For the entire cohort, the median OS from the start of second-line chemotherapy was 12.5 months (95% CI, 10.8–14.2 months). The platinum group showed significantly better OS than the non-platinum group did (median OS: 21.5 vs. 10.5 months, HR: 0.54, 95% CI: 0.40–0.73, *p* < 0.001, Fig. 2). The 12-, 24-, and 36-month OS rates were 68.2%, 39.4%, and 33.4% in the platinum group and 39.3%, 23.6%, and 15.7% in the non-platinum group, respectively. OS from the start of the first-line CRT was also significantly longer in the platinum group than in the non-platinum group (median OS: 34.9 vs. 21.8 months, HR: 0.58, 95% CI: 0.43–0.79, *p* = 0.001). The median time interval between the start of CRT and the start of second-line chemotherapy was 8.6 months. With 8.6 months as the cut-off value, the platinum group showed significantly better OS than the non-platinum group (median OS: 21.5 vs. 8.5 months, HR: 0.38, 95% CI: 0.24–0.58, *p* < 0.001, Fig. 3A) in patients with an interval of < 8.6 months. However, there was no significant difference in OS from the start of CRT between the platinum and non-platinum groups (median OS: 20.5 vs. 12.5 months, HR: 0.69, 95% CI: 0.44–1.08, *p* = 0.09; Fig. 3B) in the subgroup with an interval of ≥ 8.6 months.

Univariate analysis identified platinum-doublet chemotherapy in the second-line setting, female sex, and clinical stage IIIA as significant predictors of better OS. Multivariate analysis showed that platinum-doublet chemotherapy as second-line therapy (HR: 0.53, 95% CI: 0.39–0.73, *p* < 0.01), female sex (HR: 0.55, 95% CI: 0.34–0.88, *p* = 0.01), clinical stage IIIA (HR: 0.67, 95% CI: 0.50–0.90, *p* < 0.01), and an interval of ≥ 8.6 months from the start of the first-line CRT to the start of second-line chemotherapy (HR: 0.74, 95% CI: 0.55–0.997, *p* = 0.048) were associated with significantly better OS from the start of second-line chemotherapy (Table 3).

**Table 3:** Univariate and multivariate analysis of covariables associated with overall survival from the start of second-line chemotherapy

Variables	Univariate	*p*-value	Multivariate	*p*-value
	HR		HR	
	(95% CI)		(95% CI)	
Second-line chemotherapy				
Platinum-doublet vs Single agent	0.54	< 0.01	0.53	< 0.01
	(0.40 to 0.73)		(0.39 to 0.73)	
Age				
≥ 75 years vs < 75 years	0.79	0.41	0.82	0.51
	(0.45 to 1.34)		(0.45 to 1.49)	
Sex				
Female vs Male	0.57	0.02	0.55	0.01
	(0.36 to 0.91)		(0.34 to 0.88)	
ECOG PS				
0/1 vs 2	2.15	0.28	2.59	0.19
	(0.53 to 8.65)		(0.63 to 10.6)	
Smoking status				
Never or former smoker vs Current smoker	0.84	0.23	0.94	0.70
	(0.63 to 1.12)		(0.70 to 1.27)	
Histology				
Adenocarcinoma vs Non-adenocarcinoma	0.98	0.87	1.14	0.38
	(0.73 to 1.30)		(0.85 to 1.54)	
Clinical stage				
IIIA vs IIIB	0.62	< 0.01	0.67	< 0.01
	(0.47 to 0.82)		(0.50 to 0.90)	
Respiratory comorbidity				
No vs Yes	0.95	0.95	0.95	0.75
	(0.71 to 1.27)		(0.70 to 1.29)	
Response to first-line chemoradiotherapy				
CR or PR vs not CR nor PR	0.94	0.71	0.94	0.72
	(0.70 to 1.28)		(0.68 to 1.30)	
Duration from the start of first-line therapy to second-line therapy				
≥ 8.6 months vs < 8.6 months	0.82	0.16	0.74	0.048
	(0.62 to 1.08)		(0.55 to 0.997)	

*CI* confidence interval, *CR* complete response, *ECOG PS* Eastern Cooperative Oncology Group performance status, *HR* hazard ratio, *PR* partial response

The ORR of the second-line chemotherapy, assessed according to the RECIST, was significantly higher in the platinum group than in the non-platinum group (26.1% vs. 7.0%; *p* < 0.001). In total, 67 (56.3%) patients in the platinum group and 101 (50.2%) in the non-platinum group received subsequent chemotherapy after second-line chemotherapy. OS from the start of the third-line treatment was not significant different between the platinum and the non-platinum groups (median OS: 12.7 vs. 8.7 months, HR: 0.78, 95% CI: 0.52–1.17, *p* = 0.24).

### Survival outcome in patients with an interval of ≥ 3 months between first-line CRT and second-line chemotherapy

Among patients with an interval of ≥ 3 months between first-line CRT and second-line chemotherapy, the platinum group showed significantly better OS than the non-platinum group did (median OS: 20.5 vs. 10.5 months, HR: 0.57, 95% CI: 0.40–0.80, *p* = 0.001). The 12-month OS rate was 70.5% in the platinum group and 38.7% in the non-platinum group. The ORR of second-line chemotherapy, assessed according to the RECIST, was significantly higher in the platinum group than in the non-platinum group (23.9% vs. 7.3%; *p* < 0.001).

## Discussion

The optimal regimen for patients with relapsed NSCLC previously treated with platinum-based CRT remains unclear. This retrospective cohort study found that platinum-doublet chemotherapy was an effective treatment modality in the second-line setting in patients with unresectable stage III NSCLC previously treated with platinum-based CRT, yielding a significantly good response and survival.

These findings were consistent with the findings from previous studies on small-cell lung cancer (SCLC) and ovarian cancer. Re-administration of platinum-based agents with etoposide and irinotecan prolonged the OS in patients with platinum-sensitive relapsed SCLC with a treatment-free interval of ≥ 90 days [17]. The treatment-free interval has been reported as a relevant prognostic factor and an important predictor of the probability of response to second-line chemotherapy in patients with SCLC [18]. In patients with ovarian cancer, a platinum-free interval of ≥ 6 months has been the most widely used and accepted clinical surrogate marker to predict chemotherapy response and prognosis [19–22]. Thus, in both SCLC and ovarian cancer, the length of the platinum-free interval is considered associated with platinum sensitivity and good prognosis. In contrast, this study did not show a contribution of the platinum-free interval to the efficacy of platinum re-administration. In advanced NSCLC patients treated with a platinum agent in the second-line setting, the platinum-free interval was not associated with a survival benefit [23–25]. Arrieta et al. reported that a treatment-free interval of ≥ 9 months was associated with a long PFS in patients receiving platinum-doublet chemotherapy in the second-line setting [26]. Therefore, the impact of the platinum-free interval on the efficacy of platinum re-administration is still unclear. However, a long platinum-free interval may be an independent prognostic factor rather than an indicator of platinum sensitivity in patients with stage III NSCLC. Larger studies are warranted to evaluate the association between platinum sensitivity and the platinum-free interval in patients with stage III NSCLC.

There have been few studies on second-line treatment after platinum-based CRT in patients with stage III NSCLC. In 10 studies suggesting the efficacy of platinum-based therapy as a second-line treatment [13], 0–66.7% of all patients had stage III NSCLC and 0–36.7% of all patients had been treated with CRT [23–32]. With respect to patients with stage III NSCLC previously treated with platinum-based CRT, only a small retrospective single-institution study showed that the efficacy of platinum-based chemotherapy was equivalent to that of docetaxel in relapsed patients, with an ORR of 31.2% (5/16) and an OS of 28.0 months [33]. There was no difference in patient characteristics such as age, sex, and PS between the two groups in the study, which is consistent with our study. To the best of our knowledge, our study is the largest to evaluate the efficacy of platinum-based chemotherapy in patients with relapsed stage III NSCLC after platinum-based CRT. Our study showed numerically longer survival in stage III NSCLC patients treated with second-line platinum-based CRT (ORR, 26.1%; OS, 21.5 months) than previous reports did, which mostly included stage IV patients (ORR, 27.5%; OS, 8.7 months) [13].

Durvalumab has been recently approved for maintenance therapy following platinum-based CRT in patients with stage III NSCLC with no progression after CRT [8, 9]. However, approximately 30% of patients receiving platinum-based CRT are not eligible for maintenance therapy with durvalumab in the real-world setting [34]. Moreover, many patients still show disease progression, with a 12-month PFS rate of approximately 50–60%, and consequently need a second-line treatment after durvalumab administration [8, 9]. Currently, the combination of platinum-based chemotherapy and PD-L1/PD-1 inhibitors [35–38] or the combination of docetaxel and angiogenesis inhibitors [39, 40] is also one of the options for second-line treatment after CRT. However, whether to use platinum-based chemotherapy or single-agent chemotherapy as a second-line treatment remains a fundamental clinical question even now that CRT followed by durvalumab has become the standard treatment. Therefore, the results of our study can be applied even for patients treated with current standard treatment.

This study has some limitations. First, because the data were retrospectively extracted from a registry and not from clinical trials, assessments were probably incomplete and not performed at fixed intervals. Second, this registry did not contain data on the date of disease progression, patient characteristics at the start of second-line chemotherapy, and subsequent cancer therapies. Although it was difficult to fully exclude selection bias, there was no difference in several recognized prognostic factors between the platinum and non-platinum groups in our study as well as in previous studies. These factors should be considered in future research by propensity score matching or other means. Third, details of chemotherapy, including types of agents, treatment cycles, and dosage at each stage, were also unknown. According to the Japanese guidelines, cisplatin plus docetaxel, carboplatin plus paclitaxel, cisplatin plus vinorelbine, and cisplatin plus S-1 are frequently used with concurrent radiotherapy for the first-line treatment of stage III NSCLC patients in the real-world setting. There are no significant differences in the effectiveness of these regimens [34]. Fourth, because there was no information about the reasons for the termination of the first-line therapy, we did not know the number of discontinuations due to disease progression and adverse events. In our study, the interval between first-line CRT and second-line chemotherapy was significantly shorter in the platinum group than in the non-platinum group, but the OS was reversed. The reasons for the shorter interval between first-line CRT and second-line chemotherapy in the platinum group may include not only disease progression but also discontinuation due to adverse events and consolidation therapy counted as a second-line therapy. Therefore, we analyzed subgroup with a duration of ≥ 3 months from the first-line CRT to second-line chemotherapy to exclude as many patients as possible whose platinum-based CRT was terminated prematurely due to adverse events or because of consolidation chemotherapy counted as a second-line chemotherapy. The analysis of this subgroup also showed that the platinum group had significantly better OS than the non-platinum group.

## Conclusions

Platinum-doublet chemotherapy in the second-line setting yields significantly longer survival than single-agent chemotherapy in patients with unresectable stage III NSCLC previously treated with platinum-based CRT. Given the lack of standardized second-line treatments for stage III NSCLC patients showing relapse after platinum-based CRT, our results indicate that platinum re-administration is a promising treatment option for these patients. Although durvalumab maintenance therapy after platinum-based CRT is the current standard, our results are still meaningful for patients receiving durvalumab.

## Data Availability

The datasets generated during and analyzed during the current study are not publicly available due to participants of this study did not agree for their data to be shared publicly but are available from the corresponding author on reasonable request.

## Abbreviations

CIs: Confidence intervals
CRT: Chemoradiotherapy
CT: Computed tomography
ECOG PS: Eastern Cooperative Oncology Group performance status
EGFR: Epidermal growth factor receptor
HRs: Hazard ratios
JJCLCR: Japanese Joint Committee of Lung Cancer Registry
NSCLC: Non-small-cell lung cancer
ORR: Objective response rate
OS: Overall survival
PFS: Progression-free survival
RECIST: Response Evaluation Criteria in Solid Tumors
SCLC: Small-cell lung cancer
